# Evaluation of an Affibody-Based Binder for Imaging of Immune Check-Point Molecule B7-H3

**DOI:** 10.3390/pharmaceutics14091780

**Published:** 2022-08-25

**Authors:** Maryam Oroujeni, Ekaterina A. Bezverkhniaia, Tianqi Xu, Yongsheng Liu, Evgenii V. Plotnikov, Ida Karlberg, Eva Ryer, Anna Orlova, Vladimir Tolmachev, Fredrik Y. Frejd

**Affiliations:** 1 Department of Immunology, Genetics and Pathology, Uppsala University, 751 85 Uppsala, Sweden; 2 Affibody AB, 171 65 Solna, Sweden; 3 Research Centrum for Oncotheranostics, Research School of Chemistry and Applied Biomedical Sciences, Tomsk Polytechnic University, 634050 Tomsk, Russia; 4 Scientific and Research Laboratory of Chemical and Pharmaceutical Research, Siberian State Medical University, 634050 Tomsk, Russia; 5 Department of Medicinal Chemistry, Uppsala University, 751 83 Uppsala, Sweden

**Keywords:** B7-H3, affibody molecule, technetium-99m (^99m^Tc), AC12-GGGC, SKOV-3 xenograft, SPECT/CT imaging

## Abstract

Radionuclide molecular imaging could provide an accurate assessment of the expression of molecular targets in disseminated cancers enabling stratification of patients for specific therapies. B7-H3 (CD276) is a transmembrane protein belonging to the B7 superfamily. This protein is overexpressed in different types of human malignancies and such upregulation is generally associated with a poor clinical prognosis. In this study, targeting properties of an Affibody-based probe, AC12, containing a -GGGC amino acid sequence as a chelator (designated as AC12-GGGC) labelled with technetium-99m (^99m^Tc) were evaluated for imaging of B7-H3-expressing tumours. AC12-GGGC was efficiently labelled with ^99m^Tc. [^99m^Tc]Tc-AC12-GGGC bound specifically to B7-H3 expressing cells in vitro with affinities in nanomolar range. In mice bearing B7-H3-expressing xenografts, [^99m^Tc]Tc-AC12-GGGC showed tumour uptake of 2.1 ± 0.5 %ID/g at 2 h after injection. Its clearance from blood, normal organs and tissues was very rapid. This new targeting agent, [^99m^Tc]Tc-AC12-GGGC, provided high tumour-to-blood ratio already at 2 h (8.2 ± 1.9), which increased to 11.0 ± 0.5 at 4 h after injection. Significantly (*p* < 0.05) higher tumour-to-liver and higher tumour-to-bone ratios at 2 h in comparison with 4 h after injection were observed. Thus, [^99m^Tc]Tc-AC12-GGGC could be a promising candidate for further development.

## 1. Introduction

B7-H3 (CD276) is a 316 amino acid long type I transmembrane protein belonging to the B7 ligand family of immune checkpoint molecules [1]. Structurally, B7-H3 exists in two isoforms, named 4Ig-B7-H3 and 2Ig-B7-H3, determined by its extracellular domain. In mice, the extracellular domain comprises a single pair of immunoglobulin variable (IgV)-like and immunoglobulin constant (IgC)-like domains, while in humans, B7-H3 consists of one or two identical pairs [2,3,4]. The molecular weight of B7-H3 protein moiety is approximately 45–66 kDa, while that of the glycosylated 4Ig-hB7-H3 isoform is approximately 100 kDa [5].

The exact biological role of B7-H3 is controversial but several studies have described the role of B7-H3 as either co-stimulatory or co-inhibitory in T cell-mediated adaptive immunity [6]. B7-H3 is mostly expressed on the surface of T and B cells [1,7]. It has a low level of expression in most normal organs and tissues, but is overexpressed in a wide variety of cancers, such as prostate [8,9], renal cell carcinoma [10], urothelial cell carcinoma [11], ovarian cancer [12], glioblastoma [13], osteosarcoma [14], pancreatic cancer [15], neuroblastoma [16], diffuse intrinsic pontine glioma [17] and mesothelioma [18].

In malignant tissues, B7-H3 inhibits tumour antigen-specific immune responses, leading to a protumourigenic effect. B7-H3 also has nonimmunologic protumourigenic functions, such as promoting migration and invasion, angiogenesis, chemoresistance, endothelial-to-mesenchymal transition and affecting tumour cell metabolism. Remarkably, overexpression of B7-H3 is associated with increased tumour aggressiveness, poor prognosis, and resistance in many cancers [9,14,19,20,21,22,23,24,25].

It has been observed that there is a correlation between B7-H3 level in serum of cancerous patients and clinicopathologic variables, suggesting the use of B7-H3 as a noninvasive target for diagnosis, prognosis, and/or treatment applications [26]. Recent clinical studies concerning antibody-based immunotherapy have enabled the identification of clinically relevant tumour antigens that could be used as targets in solid tumours. Among them, B7-H3 represents an attractive target for antibody-based immunotherapy. Some radiolabelled antibody-based probes for targeting B7-H3–expressing tumours have been developed [27,28,29]. These studies have demonstrated an effective anti-tumour activity and an acceptable safety of these probes in preclinical models. A phase I clinical trial has demonstrated safety and efficacy of an antibody-based tracer in patients [29].

Nuclear medicine imaging modalities, such as single-photon emission computed tomography (SPECT) and positron-emission tomography (PET), are widely used in preclinical and clinical applications. Although, the use of the PET modality provides higher specificity and sensitivity of imaging in comparison to SPECT, PET has some drawbacks such as limited availability [30]. Moreover, the progress in detectors and software development makes SPECT/CT an increasingly attractive imaging modality in clinics and allows for less costly cameras than PET scanners [31].

The use of generator-produced radionuclide technetium-99m (^99m^Tc) makes the process of radiopharmaceutical production appreciably cheaper without the necessity of cyclotron installation. Additionally, high availability, optimal half-life (T_1/2_ = 6 h) and low absorbed-dose burden to the patient are some of the advantages of this radionuclide. Taking this into account, ^99m^Tc is an attractive radionuclide for clinical imaging [32].

Imaging probes based on antibodies have shown a slow extravasation in tumour and these targeting probes are cleared slowly from blood circulation. This causes a low imaging contrast (even several days after injection) and an elevated dose burden to the patient [33,34,35]. The use of small imaging probes such as engineered scaffold proteins (ESPs) has been introduced as a suitable alternative. Compared to antibodies, ESPs have higher extravasation rate in tumour and faster clearance from blood and non-targeted tissues, which resulted in a reduction in the imaging time to a few hours after injection and increased imaging contrast (increased sensitivity) [36]. One of the promising ESPs for radionuclide imaging are Affibody molecules [37,38]. Affibody molecules are based on a 58-amino acid cysteine-free scaffold, which can be produced either synthetically or in bacteria by the use of recombinant DNA technology [37]. Small size (6–7 kDa), high thermal and chemical stability (withstanding 90 °C and broad range of pH without denaturation), site-specific radiolabelling and high affinity are of the features of these molecules in comparison with monoclonal antibodies, making them promising targeting agents against different molecular targets [37]. Taking this into account, it has provided a strong motivation for application of Affibody-based imaging probes in the clinic [39,40].

An Affibody-based binder AC12 produced by cellular-based selections aid yeast-display was recently introduced [41]. Efficacy of Affibody-based tracer for ultrasound molecular imaging of vascular B7-H3 in breast cancer has been already proved [42].

**Figure 1:**
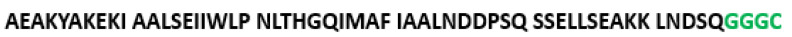
The sequence of amino acids in AC12-GGGC. For coupling ^99m^Tc to AC12, the sequence APK at C-terminus of parental AC12 Affibody molecule [41] was replaced by the sequence GGGC.

For coupling ^99m^Tc to the proteins and/or peptides, a chelator conjugation is necessary. The feasibility of the use of peptide-based cysteine containing chelators for coupling of ^99m^Tc and the influence of their composition and order of amino acids in these chelators have been described in several studies [43,44,45]. Amongst evaluated chelators, the –GGGC (triglycine-cysteine) chelator was selected as a potential candidate for labelling of Affibody molecules with ^99m^Tc because the use of this composition of amino acids at binding site in Affibody molecules provides high tumour uptake and a low retention of activity in the kidneys based on previous studies [44,45]. The structure of AC12-GGGC has been shown in Figure 1.

The goal of this study was to evaluate targeting properties of the AC12 Affibody molecule labelled with ^99m^Tc using the peptide-based cysteine containing –GGGC chelator (designated as AC12-GGGC). For this purpose, AC12-GGGC was produced and characterised. The labelling of AC12-GGGC Affibody molecule with ^99m^Tc was optimized using transchelation from gluconate. Stability and in vitro properties of [^99m^Tc]Tc-AC12-GGGC were tested. In vivo targeting properties of AC12-GGGC labelled with ^99m^Tc was studied in mice bearing B7-H3-expressing SKOV-3 xenografts. nanoSPECT/CT imaging was performed to confirm feasibility of imaging of B7-H3-expressing tumours using [^99m^Tc]Tc-AC12-GGGC and also to confirm in vivo specificity of this imaging agent.

## 2. Materials and Methods

### 2.1. General

High quality Milli-Q water was used to prepare all buffer solutions. ^99m^Tc was obtained as pertechnetate by elution of an Ultra TechneKow generator (Mallinckrodt, Petten, The Netherlands) with sterile 0.9% sodium chloride (Mallinckrodt, Petten, the Netherlands). The Cyclone Storage Phosphor system (Perkin-Elmer, Wellesley, MA, USA) was used for the quantitative measurement of radioactivity distribution in instant thin-layer chromatography strips. The activity from cell and animal samples was measured using an automated gamma-spectrometer equipped with a 3-inch NaI (TI) well detector (2480 Wizard, Wallac, Turku, Finland). Radioactivity for labelling and injection formulation was measured using a dose calibrator VDC-405 (Veenstra Instruments BV, Joure, The Netherlands) equipped with an ionization chamber.

In vitro cell studies were performed using the B7-H3-expressing the ovarian cancerSKOV-3 and the breast cancer BT-474 cell lines, obtained from the American Type Culture Collection (ATCC). Ramos lymphoma cells (ATCC) were used to establish B7-H3-negative xenografts. Cells were cultured in RPMI medium (Flow Laboratories, Irvine, UK) supplemented with 10% of fetal calf serum (20% of fetal calf serum for BT-474), 2 mM of L-glutamine, 100 IU/mL of penicillin, and 100 mg/mL of streptomycin.

Statistical analysis was performed using GraphPad Prism software version 5.00 for Windows (GraphPad Software, San Diego, CA, USA). A two-tailed unpaired t-test was used for comparison of the two sets of data. The difference was considered significant when the *p* value was less than 0.05.

### 2.2. Production, Purification, and Characterization of Novel Anti-B7-H3 Affibody Molecules

The Affibody molecule AC12-GGGC was produced by solid phase peptide synthesis using the Fmoc/tBu strategy. The synthesised Affibody molecule was purified by preparative reversed phase high-performance liquid chromatography, buffer was exchanged to an acetate-containing buffer and finally lyophilized in aliquots of about 4 mg. The lyophilized Affibody molecule was stored at −20 °C. This work was performed as fee-for-service by the contract manufacturer Bachem (St Helens, UK).

For the Radiolabelling, lyophilized Affibody molecule AC12-GGGC was weighted and dissolved in PBS (Corning, Glendale, AZ, USA) supplemented with 2 mM EDTA (Corning, Glendale, AZ, USA) to a final concentration of 2.0 mg/mL, taking the peptide content of 94.7% in consideration. The solubilized Affibody molecule was aliquoted in 50 µL aliquots (100 µg) and stored at −80 °C.

Affibody molecule AC12-GGGC, diluted to 0.5 mg/mL in PBS (Corning, Glendale, AZ, USA) supplemented with 2 mM EDTA (Corning, Glendale, AZ, USA), was characterized by Circular Dichroism (Jasco J-810 spectropolarimeter, Jasco Scandinavia AB, Mölndal, Sweden). A Circular Dichroism (CD) spectrum at 250–195 nm was performed at 20 °C. A new CD spectrum was performed after a variable temperature measurement (VTM) at 221 nm, 20 °C to 90 °C, to analyse refolding of AC12-GGGC after thermal denaturation.

An IEF gel analysis was performed according to manufacturer’s instructions to determine the isoelectric point of the molecule. Samples were loaded on a NovexTM pH 3-10 Protein Gel (ThermoFisher, Waltham, MA, USA) together with an IEF Marker 3-10 (Serva, Heidelberg, Germany). The gel was fixed in 12% TCA (Merck KGaA, Darmstadt, Germany), stained in InstantBlueR Coomassie Protein Stain (Abcam, Cambridge, UK) and destained in MilliQ water before image was captured using Gel DocTM EZ System, ImageLab software (BioRad, Hercules, CA, USA).

The purity of AC12-GGGC was determined by reversed phase ultra-high-performance liquid chromatography (RP-UPLC) on an Acquity UPLC CSH-C18, 1.7 µm, 2.1 × 150 mm column (Waters, Milford, MA, USA). Elution was performed by a linear gradient of acetonitrile from 10% to 60% in TFA for 24 min. The flow rate was 0.2 mL/min, the temperature 65 °C and the 220 nm signal was monitored for integration.

The HER2-binding Affibody molecule ZHER2:41071 having the same GGGC chelator, which was used for in vivo comparison, was produced as previously described [45].

### 2.3. Radiolabelling and In Vitro Stability

The AC12-GGGC Affibody molecule was site-specifically labelled with ^99m^Tc using a lyophilized kit. A freeze-dried labelling kit containing 75 µg of tin (II) chloride dihydrate (Fluka Chemika, Buchs, Switzerland), 5 mg of gluconic acid sodium salt (Celsus Laboratories, Geel, Belgium), and 100 µg of ethylenediaminetetraacetic acid tetra sodium salt (Sigma-Aldrich, Munich, Germany) was prepared for labelling of Affibody molecule with ^99m^Tc, as described earlier [46].

Radiolabelling of AC12-GGGC was performed by adding the contents of the freeze-dried kit, dissolved in 120 µL degassed PBS, to 100 µg of the AC12-GGGC. 80 µL (200–300 MBq) of the generator eluted ^99m^Tc-pertechnetate was added to the reaction mixture and the vial was degassed to protect the mixture from oxidation. The reaction vial was thoroughly vortexed and incubated at 90 °C for 1 h. Radiochemical yield was analysed using instant thin layer chromatography (ITLC-SG) (Agilent Technologies, Santa Clara, CA, USA) developed with PBS (Affibody: Rf = 0.0, other forms of ^99m^Tc: Rf = 1.0). The reduced hydrolyzed technetium colloid (RHT) in the mixture was measured using pyridine:acetic acid:water (10:6:3) as the mobile phase (^99m^Tc colloid: Rf = 0.0, other forms of ^99m^Tc and radio-labelled Affibody molecule: Rf = 1.0). Since the radiochemical yield was over 95%, no further purification was performed. ZHER2:41071 Affibody molecule for in vivo comparison was labelled in the same way. The radiochemical purity of [^99m^Tc]Tc-ZHER2:41071 was determined using ITLC-SG using the same protocol as for analysis of [^99m^Tc]Tc-AC12-GGGC. The use of this protocol for analysis of [^99m^Tc]Tc-ZHER2:41071 was cross-validated by radio-HPLC earlier [45]. The radiochemical purity was over 97 % and no further purification was performed.

To cross-validate radio-ITLC data further, reverse phase-HPLC was conducted using an EliteLaChrom system (Hitachi, VWR, Darmstadt, Germany) consisting of an L-2130 pump, a UV detector (L-2400), and a radiation flow detector (Bioscan, Washington, DC, USA) coupled in series was used. A purity analysis of ^99m^Tc-labelled compound was performed using an analytical column (Vydac RP C18 column, 300 Å; 3 × 150 mm; 5 µm). HPLC conditions were as follows: A = 10 mM TFA/H2O, B = 10 mM TFA/acetonitrile, UV-detection at 280 nm, gradient elution: 0–15 min at 5% to 70% B, 15–18 min at 70% to 95% B, 19–20 min at 5% B, and a flow rate was 1.0 mL/min.

To evaluate stability, fractions of the freshly radiolabelled conjugate (10 µL, 4 µg) were incubated with an excess amount of PBS (40 µL) for 1 and 4 h at 37 °C. The test was run in triplicates. To evaluate stability in blood serum, two samples of radiolabeled [^99m^Tc]Tc-AC12-GGGC (0.15 µg) were mixed with the murine blood serum (100 µL) to mimic a concentration of the tracer in murine blood immediately after injection. Two control samples were mixed with the same amount of PBS. All samples were incubated at 37 °C during 60 min, and thereafter were passed through NAP5 columns (Cytiva, Uppsala, Sweden) to separate a high molecular weight compounds (over 5 kDa) and low molecular weight compounds (less than 5 kDa). Activities in both high- and low-molecular weight fractions were measured to calculate percentage of ^99m^Tc associated with the high molecular weight fraction (i.e., bound to Affibody molecules).

### 2.4. In Vitro Studies

Ovarian carcinoma SKOV-3 and breast carcinoma BT-474 cell lines were used for cell studies as B7-H3-positive cell lines. Ramos lymphoma cell line was used as a B7-H3-negative control. A B7-H3 specific antibody (R&D systems mouse IgG1, #MAB1027) was used to rank receptor expression levels of different cell lines by titration and quantification of expression using BD Quantibrite beads. Results show high expression level for SKOV-3, intermediate for BT-474 and very low or no expression of B7-H3 for Ramos cells. The B7-H3 expression levels were estimated to be 68,000, 45,000 and 250 receptors per cell for SKOV-3, BT-474 and Ramos, respectively (Figure S1). Cells were seeded in cell-culture dishes (35 mm in diameter) with a density of 10^6^ cells/dish. A set of three dishes was used for in vitro binding specificity.

For in vitro binding specificity of the conjugate, cells in three control dishes were pre-saturated with 200-fold excess of non-labelled AC12-GGGC 15 min before addition of the labelled conjugate. The cells in both blocked and nonblocked dishes were incubated with a labelled conjugate (10 nM) in a humidified incubator (5% CO_2_, 37 °C) for 1 h. The medium was discarded, the cells were washed with cold serum-free medium before trypsin–EDTA solution (0.5 mL per dish) was added, and cells were additionally incubated for 10 min. Detached cells were diluted with 0.5 mL of complete medium, re-suspended, and transferred to fraction tubes. The radioactivity of cells was measured using an automated gamma counter and the cell-bound radioactivity was calculated.

To evaluate the affinity of binding of the radiolabelled conjugate to B7-H3 target, kinetics of binding of ^99m^Tc-labelled AC12-GGGC to and its dissociation from SKOV-3 cells were measured using a LigandTracer Yellow instrument (Ridgeview Instruments AB, Vänge, Sweden), as previously described [47]. SKOV-3 cells were seeded on a local area of a cell culture dish (89 mm in diameter, NunclonTM, NUNC A/S, Roskilde, Denmark). The measurements were performed at room temperature to prevent internalization. Uptake curves were recorded at 2, 6, and 18 nM of ^99m^Tc-labelled AC12-GGGC. Thereafter, the radioactive medium was withdrawn, fresh complete non-radioactive medium was added, and the dissociation curve was recorded. Analysis was performed in duplicates. The data were analysed using the Interaction Map software (Ridgeview Diagnostics AB, Uppsala, Sweden) to calculate the association rate, the dissociation rate, and the dissociation constant at equilibrium (KD). The basics of the InteractionMap analysis was described by Altschuh and co-workers [48]. 

### 2.5. In Vivo Studies

Animal experiments were performed in accordance with the national legislation for work with laboratory animals. Approval was granted by the Ethical Committee for Animal Research in Uppsala (ethical permission C4/16, decision from 26 February 2016). Groups of four mice per data point were used for these experiments.

Biodistribution of [^99m^Tc]Tc-AC12-GGGC was studied in female NMRI mice 4 h after injection and it was compared head-to-head with the biodistribution of [^99m^Tc]Tc-ZHER2:41071 Affibody molecule. A group of non-tumour bearing mice (average weight = 32 ± 1 g) were injected with 3 µg of [^99m^Tc]Tc-AC12-GGGC (60 kBq, 100 µL in PBS) into the tail vein. As a comparison, the second group was injected with [^99m^Tc]Tc-ZHER2:41071 (3 µg/ 60 kBq, 100 µL in PBS). After 4 h, mice were euthanized by overdosing of anaesthetic solution (20 μL of solution per gram of body weight: ketamine, 10 mg/mL; Xylazine, 1 mg/mL). This was followed by a heart puncture, and blood samples were collected. Organs and tissues were collected and weighed. The organ radioactivity was measured using a gamma spectrometer along with three standards and empty syringes for each animal. Uptake values for organs were calculated as the percentage-injected dose per gram tissue (%ID/g). To analyze radiometabolites of [^99m^Tc]Tc-AC12-GGGC in urine, this compound (8.4 µg, 18 MBq) was injected in tail veins of two NMRI mice. Immediately before injection, [^99m^Tc]Tc-AC12-GGGC was analyzed using radio-HPLC as described in Section 2.3, Radiolabeling and In Vitro Stability. The mice were euthanized by overdosing of anesthetic solution 30 min after injection. Urine was taken from bladders using syringes and analyzed using radio-HPLC.

Biodistribution and targeting properties of [^99m^Tc]Tc-AC12-GGGC were evaluated in BALB/C nu/nu mice bearing B7-H3-positive SKOV-3 xenografts. To establish xenografts, SKOV-3 cells (10^7^ cells per mouse) were subcutaneously implanted on the right hind leg of female BALB/c nu/nu mice. To check in vivo specificity, B7-H3-negative Ramos xenografts were used. 5 × 10^6^ Ramos cells were subcutaneously implanted on the left hind leg of female BALB/c nu/nu in four mice. The experiments were performed two weeks after cell implantation. The average animal weight was 20.5 ± 1.0 and 21.0 ± 0.9 g for mice bearing SKOV-3 and Ramos xenografts, respectively. The average tumour weight was 0.11 ± 0.10 and 0.11 ± 0.09 g for SKOV-3 and Ramos xenografts, respectively. The biodistribution of [^99m^Tc]Tc-AC12-GGGC was measured 2 and 4 h after injection in mice bearing SKOV-3 xenografts. Two groups of tumour bearing mice were injected with [^99m^Tc]Tc-AC12-GGGC (3 µg, 60 kBq, 100 µL in PBS) into the tail vein. To test B7-H3-specificity of [^99m^Tc]Tc-AC12-GGGC accumulation in tumours, one group of animals bearing B7-H3-negative Ramos xenografts was injected with [^99m^Tc]Tc-AC12-GGGC (3 µg, 60 kBq, 100 µL in PBS), and the biodistribution was measured 4 h after injection. The measurement of biodistribution in tumour-bearing mice was performed in the same way as in NMRI mice (see above).

To confirm the biodistribution results, a small animal SPECT/CT imaging was performed. One SKOV-3 bearing mouse and one Ramos bearing mouse were intravenously injected with [^99m^Tc]Tc-AC12-GGGC (3 µg,6 MBq, 100 µL in PBS). The mice were imaged at 4 h after injection using a nanoSPECT/CT scanner (Mediso Medical Imaging Systems, Budapest, Hungary). The mice were euthanized by CO_2_ asphyxiation immediately before being placed in the camera. The computed tomography (CT) acquisition was carried out at the following parameters: energy peak of 50 kV, 670 µA, 480 projections, and 2.29-min acquisition time. SPECT acquisition was performed at the following parameters: ^99m^Tc energy peak of 140 keV, window width of 20%, matrix of 256 × 256, and acquisition time of 1 h. CT images were reconstructed in real-time using Nucline 2.03 Software (Mediso Medical Imaging Systems, Budapest, Hungary). SPECT raw data were reconstructed using TeraTomo™ 3D SPECT reconstruction technology.

## 3. Results

### 3.1. Production, Purification, and Characterization of Novel Anti-B7-H3 Affibody Molecules

The Lyophilized AC12-GGGC Affibody molecule was dissolved in PBS supplemented with 2 mM EDTA and the purity was determined to be 93 % by reversed phase ultra-high-performance liquid chromatography (RP-UPLC). By IEF gel analysis, the isoelectric point (pI) was determined to be 4.6.

**Figure 2:**
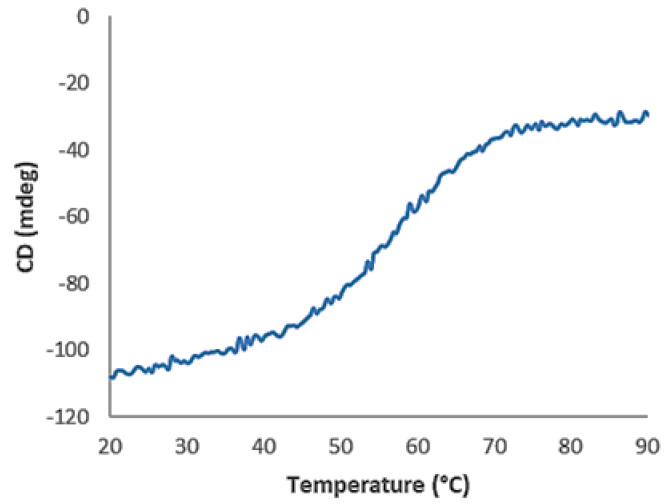
The CD (in mdeg) is plotted as a function of temperature. The Tm value was determined to 55 °C at maximum slope.

**Figure 3:**
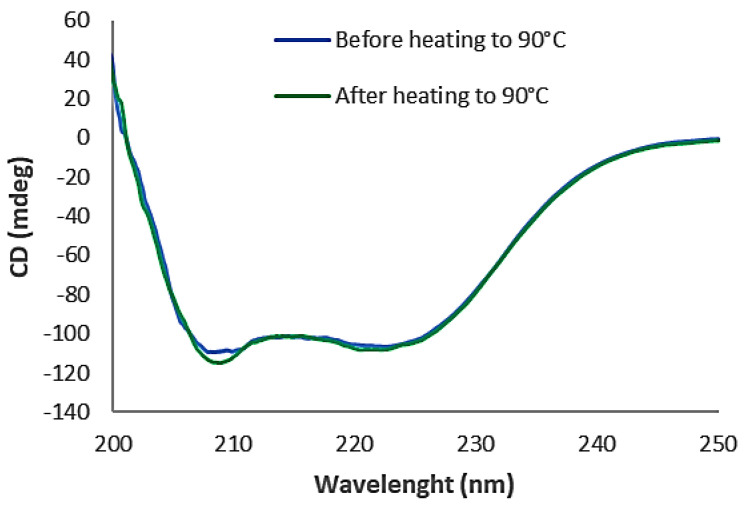
The CD (in mdeg) is plotted as a function of wavelength. Folding seems to be reversible after heating the molecules to 90 °C.

Circular dichroism (CD) analysis showed that AC12-GGGC has an α-helix secondary structure and refolds with maintained overall structure after thermal denaturation. The melting temperature (Tm) was determined to 55 °C (Figure 2). A good overlap between the two CD spectra was obtained before and after thermal denaturation at 90 °C (Figure 3).

### 3.2. Radiolabelling and In Vitro Stability

**Figure 4:**
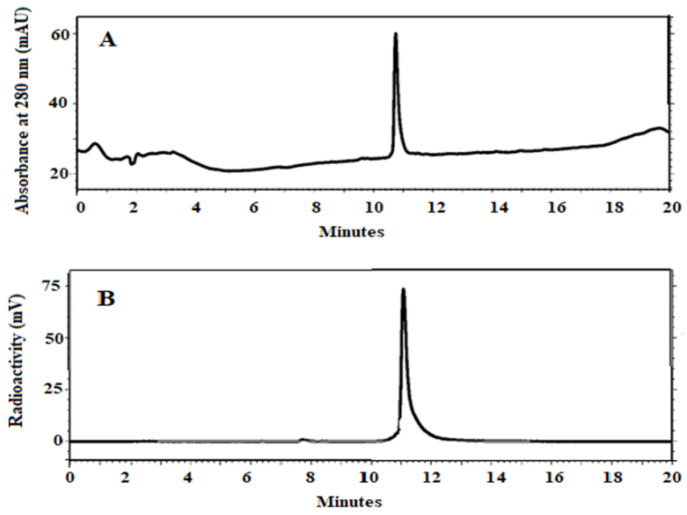
Characterization of anti-B7-H3 Affibody molecule. Reversed-phase HPLC chromatograms of non-labelled (**A**) AC12-GGGC and radiochromatogram of (**B**) [^99m^Tc]Tc-AC12-GGGC. The retention times (Rt) are expressed in minutes.

The anti-B7-H3 AC12-GGGC Affibody molecule was successfully labelled with ^99m^Tc, with labelling yield 96.4 ± 1.5% (*n* = 7). Since the radiochemical yield was over 95%, and RHT was less than 5%, no further purification using NAP-5 was performed for in vitro and in vivo studies. The specific activity of ^99m^Tc-labelled conjugates was 3 MBq/µg (molar activity 18.9 GBq/µmol). [^99m^Tc]Tc-AC12-GGGC conjugate was stable during incubation at 37 °C for 1 and 4 h in the presence of excess of phosphate-buffered saline (PBS). Less than 8% release of ^99m^Tc was observed up to 4 h. To cross-validate radio-iTLC data, radio-HPLC analysis was performed. According to radio-HPLC radiochromatogram (Figure 4B), the retention time of [^99m^Tc]Tc-AC12-GGGC was 11.1 min. The retention time of the labelled probe (Figure 4B) was the same as the non-labelled one (ultraviolet detector) (Figure 4A).

After 1-h incubation of [^99m^Tc]Tc-AC12-GGGC in murine serum at 37 °C, 94.8 ± 0.2% of technetium was associated with the high molecular weight fraction (molecular weight over 5 kDa). In control samples, which were diluted in PBS to the same concentration and incubated in the same condition, 94.6 ± 0.1% if technetium was associated with the high molecular fraction.

### 3.3. In Vitro Studies

**Figure 5:**
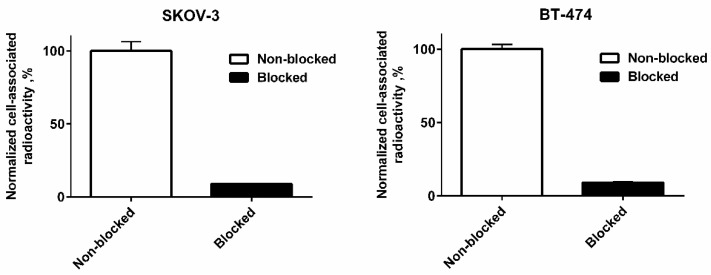
In vitro binding specificity of [^99m^Tc]Tc-AC12-GGGC on SKOV-3 and BT-474 cell lines. For the pre-saturation of B7-H3, a 200-fold molar excess of a non-labelled Affibody molecule was added before adding the labelled conjugate. Data are normalized to the average value of cell-associated radioactivity for non-blocked cells for each cell line. The data are presented as an average value from three samples ± SD.

In vitro B7-H3-binding specificity of [^99m^Tc]Tc-AC12-GGGC conjugate was tested using a saturation experiment. The binding was significantly (*p* < 5 × 10^−5^) decreased when the cells were pre-saturated with the excess amount of non-labelled anti-B7-H3 Affibody molecule (Figure 5), demonstrating that the binding was B7-H3-mediated.

**Figure 6:**
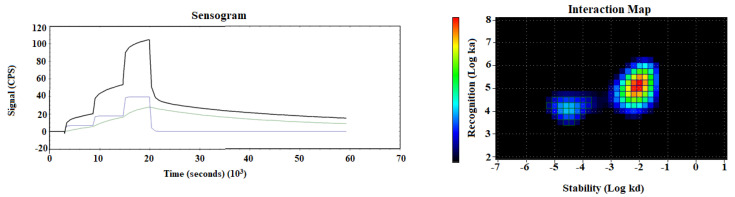
LigandTracer sensorgram and InteractionMap of [^99m^Tc]Tc-AC12-GGGC binding to SKOV-3 cells. Input data were obtained from LigandTracer measurement of cell-bound activity during association of labelled conjugate to- and dissociation from SKOV-3 cells. Binding was measured at three different concentrations: 2, 6, and 18 nM. Measurement was performed in duplicates.

The binding kinetics of [^99m^Tc]Tc- AC12-GGGC to SKOV-3 cells were investigated in real-time using a LigandTracer Yellow instrument. The results are presented in Figure 5 and Table 1. According to the LigandTracer measurements, the best fit of the binding of the conjugate to the SKOV-3 cells was achieved using a 1:2 model (Figure 6). Interaction Map calculations showed affinities in nanomolar range, a minor but strong interaction with K_D1_ = 1.9 ± 0.8 nM (%weight = 20) and a major but weaker interaction with K_D2_ = 68.8 ± 7.4 nM (%weight = 60). The corresponding equilibrium dissociation constants (K_D_) values were shown in Table 1.

### 3.4. In Vivo Studies

**Figure 7:**
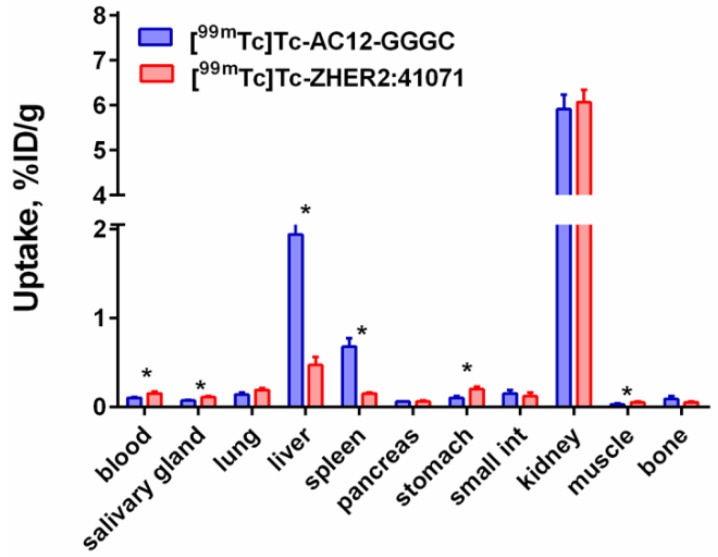
Comparative biodistribution of [^99m^Tc]Tc-AC12-GGGC and [^99m^Tc]Tc-ZHER2:41071 Affibody molecules in different organs and tissues of female NMRI mice at 4 h after injection. 3 µg of labelled Affibody molecule (60 kBq, 100 µL in PBS) was injected into the tail vein. Data are expressed as %ID/g and are averages from four mice ± SD. The significance indicator (*) corresponds to *p* < 0.05 in.

The results of a head-to-head comparison of biodistribution of [^99m^Tc]Tc-AC12-GGGC and [^99m^Tc]Tc-ZHER2:41071 Affibody molecules in NMRI mice 4 h after injection are presented in Figure 7. Biodistribution data demonstrated significantly (*p* < 0.005) lower blood concentration and lower uptake in all of the organs and tissues except for liver, spleen and bone for [^99m^Tc]Tc-AC12-GGGC in comparison with [^99m^Tc]Tc-ZHER2:41071. The hepatic uptake was significantly (*p* < 0.0005) higher for [^99m^Tc]Tc-AC12-GGGC (1.9 ± 0.2 %ID/g) than for [^99m^Tc]Tc-ZHER2:41071(0.5 ± 0.1 %ID/g) but the renal uptake was at the same level for both radioconjugates (5.9 ± 0.6 %ID/g and 6.1 ± 0.3 %ID/g for [^99m^Tc]Tc-AC12-GGGC and [^99m^Tc]Tc-ZHER2:41071, respectively). The results of HPLC analysis of radiometabolites of [^99m^Tc]Tc-AC12-GGGC in urine collected 30 min after injection are presented in the Figure S2. An appreciable part of activity was found in the radiochemical form, which different form the form of [^99m^Tc]Tc-AC12-GGGC.

**Figure 8:**
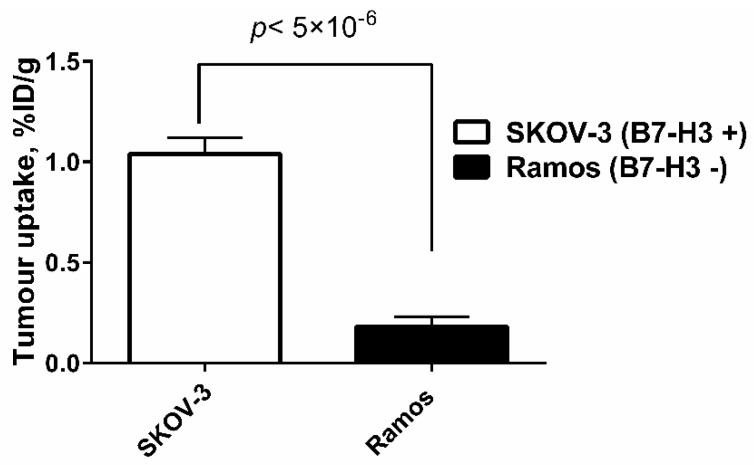
Uptake of [^99m^Tc]Tc-AC12-GGGC in SKOV-3 (B7-H3-positive) and Ramos (B7-H3-negative) xenografts at 4 h after injection. Data are expressed as %ID/g and are averages from four mice ± SD. *p*-value was obtained in unpaired *t*-test.

Experiments in nude mice-bearing human cancer xenografts demonstrated that the uptake of [^99m^Tc]Tc-AC12-GGGC in B7-H3-positive SKOV-3 xenografts was significantly (*p* < 5 × 10^−6^) higher than in B7-H3-negative Ramos xenografts 4 h after injection (Figure 8), which supports that the tumour accumulation was B7-H3-specific in vivo.

Biodistribution data and tumour-to-organ ratio of [^99m^Tc]Tc-AC12-GGGC at 2 and 4 h after injection are presented in Table 2. [^99m^Tc]Tc-AC12-GGGC showed that the tumour uptake amounted to 2.1 ± 0.5 %ID/g already at 2 h after injection. The clearance of activity from the tumour and other tissues over time was in line with the non-residualizing properties of the label. The tumour uptake decreased by a factor of two by 4 h after injection (1.0 ± 0.1 %ID/g). Moreover, the clearance from blood and normal organs and tissues such as salivary glands, lung, pancreas, stomach, muscle and bone was very rapid over time. No significant difference in renal-associated activity was observed for both time points (13.7 ± 1.8 %ID/g for 2 h p.i. compared to 10.3 ± 1.1 %ID/g for 4 h p.i.). Such a biodistribution pattern resulted in significantly (*p* < 0.05) higher tumour-to-blood at 4 h (11.0 ± 0.5) compared to 2 h (8.2 ± 1.9) after injection (Table 2). Importantly, significantly (*p* < 0.05) higher tumour-to-liver (0.7 ± 0.1 for 2 h p.i. in comparison to 0.4 ± 0.1 for 4 h p.i.) and tumour-to-bone (58.5 ± 5.0 for 2 h p.i. compared to 43.6 ± 5.7 for 4 h p.i.) ratios was observed at only 2 h after injection, which are very important for detection of hepatic and bone metastases at early time point of imaging.

**Figure 9:**
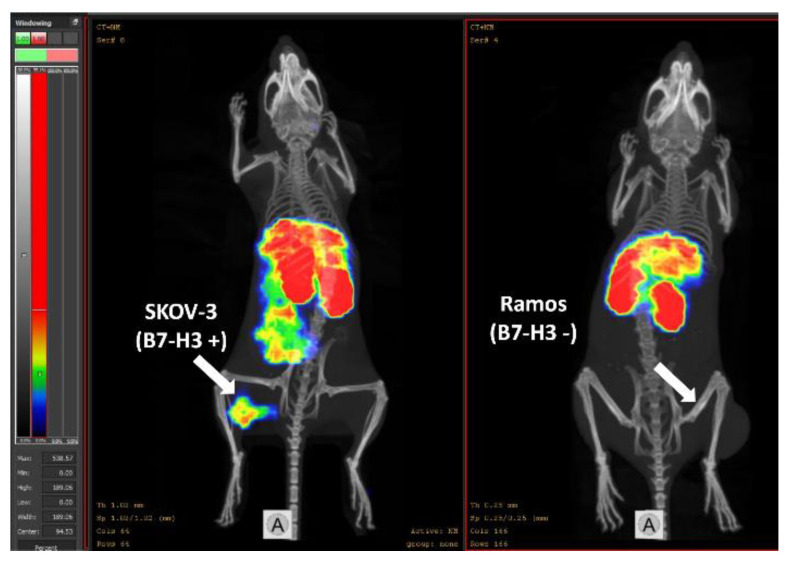
Imaging of B7-H3-positive SKOV-3 xenograft (tumour in the left mouse) and B7-H3-negative Ramos xenograft (tumour in the right mouse) in BALB/C nu/nu mice using [^99m^Tc]Tc-AC12-GGGC 4 h after injection. 3 µg of labelled Affibody molecule (6 MBq, 100 µL in PBS) was injected into the tail vein. Arrows point at tumours.

Results of nanoSPECT/CT imaging (Figure 9) demonstrated a high-contrast visualization of B7-H3 expression in B7-H3-expressing SKOV-3 tumour using [^99m^Tc]Tc-AC12-GGGC at 4 h post injection. Activity uptake in B7-H3-negative Ramos xenografts was appreciably lower than in the SKOV-3 xenograft, which confirmed B7-H3-mediated binding of this tracer in vivo. Furthermore, a high accumulation of activity was visualized in kidneys and liver, which is in agreement with the ex vivo biodistribution measurements data. In the case of a mouse with the SKOV-3 xenograft, a noticeable activity was visualized in caecum.

## 4. Discussion

The use of small scaffold proteins, such as Affibody molecules, offers an advantage in radionuclide molecular imaging compared with the use of monoclonal antibodies due to the potential of Affibody molecules to provide higher imaging contrast [49].

^99m^Tc has shown a high thiol-affinity, which enables its chelation by short peptide sequences incorporating a thiol-containing moiety. It has been shown [44,50] that in such structures, amide nitrogens of amino acids and the thiol group of cysteine form together a N3S chelator, providing a stable complex with ^99m^Tc. The use of a peptide-based cysteine-containing chelator for labelling of Affibody molecules with ^99m^Tc has advantages, such as the possibility of incorporation of chelating moiety during recombinant production without necessity of chemical conjugation. Additionally, the modification of amino acid composition of such a chelator might improve the biodistribution and targeting properties of the tracer [43]. In this study, the recently selected AC12 Affibody molecule [41] was used for targeting of the B7-H3 checkpoint molecule. According to previous studies [44,45], –GGGC could be a potential candidate for labelling of Affibody molecules with ^99m^Tc. Thus, –GGGC was incorporated at the C-terminus at AC12 Affibody molecule for labelling with ^99m^Tc. The labelling was performed with high efficiency. No release of activity from Affibody molecule was observed after 1-h incubation in murine serum at 37 °C. [^99m^Tc]Tc-AC12-GGGC binding to both HER2-expressing cell lines was highly B7-H3 specific in vitro (Figure 5). The affinity of binding to living B7-H3-expressing cells was in nanomolar range (Figure 6 and Table 1). An interesting phenomenon is the presence of two interactions of [^99m^Tc]Tc-AC12-GGGC with B7-H3 on living cells. These interactions have similar on-rate but different off-rates. The presence of different interactions might be explained by architecture of B7-H3, which is built from several identical units [5]. Epitopes, which are located on different subunits, would have the same amino acid composition, but other factor influencing binding strength, e.g., steric hindrance, might be different for different epitope locations. Thus, affinities to different epitopes would be not the same. 

A pilot experiment in normal mice (Figure 7) demonstrated that [^99m^Tc]Tc-AC12-GGGC had lower uptake in all organs and tissues except for liver, spleen and bone in comparison with the previously studied [^99m^Tc]Tc-ZHER2:41071 Affibody molecule. Our previous studies have demonstrated that even minor changes in amino acid composition in the binding site of Affibody molecules (2–3 amino acids) can appreciably influence their biodistribution profile [51,52,53]. The difference in uptake in liver and spleen and retention in blood was 2–4-fold. Most likely, this is caused by difference in off-target interactions. Thus, the difference in biodistribution of [^99m^Tc]Tc-AC12-GGGC and [^99m^Tc]Tc-ZHER2:41071 indicates an influence of different binding sites on uptake in different organs, since both radioconjugates possess the same scaffold backbone. The peptide purity of the AC12 construct was on the lower side, with 93% purity as measured in UPLC compared to 95% which is normally used as lower limit, and this may influence the distribution, but it is deemed only to a minor extent. The renal uptake of [^99m^Tc]Tc-AC12-GGGC was at the same level as the uptake of the previously studied Affibody molecule against HER2 ([^99m^Tc]Tc-ZHER2:41071), approximately 6 %ID/g. This is much lower that the renal uptake of activity after injection of Affibody molecules labelled with residualizing ^111^In or ^68^Ga labels (220–390 %ID/g) [51,52,54] but on the same level as the renal uptake of Affibody molecules with non-residualizing radiohalogen labels (4–10 %ID/g) [53,55]. The most possible explanation of the reduced renal uptake would be the non-residualizing properties of the [^99m^Tc](Tc=O)–GGGC label. The renal reabsorption of Affibody molecules causes a rapid internalization followed by intracellular degradation, and radiocatabolites of non-residualizing labels are excreted from kidneys by slow diffusion process through lysosomal and cellular membranes [38]. This is in agreement with our finding of non-Affibody-associated activity in the urine after injection of [^99m^Tc]Tc-AC12-GGGC (Figure S2). Thus, the use of a non-residualizing label should result in reduced uptake in normal organs and tissues specifically in kidneys [38]. Analysis of data from previous studies [56,57] has revealed a rapid clearance from the liver for Affibody molecules with non-residualizing properties. This would be most probably due to this phenomenon that the hepatic uptake is also associated with unspecific and high protein uptake, similar to the process in the kidneys.

The uptake of [^99m^Tc]Tc-AC12-GGGC in B7-H3-positive SKOV-3 xenografts was 6-fold higher than in B7-H3-negative Ramos xenografts (Figure 8 and Figure 9), which clearly demonstrates B7-H3-specific targeting of [^99m^Tc]Tc-AC12-GGGC in vivo. In a previous study [27], an anti-B7-H3 humanized monoclonal antibody labelled with zirconium-89 (^89^Zr-DS-5573a) was used for preclinical imaging of B7-H3 in MDA-MB-231 tumours, and showed a high tumour uptake (4.58 ± 0.69 %ID/g), with normal tissues demonstrating clearance patterns typical of a radiolabelled intact humanized antibody. Importantly, the uptake in blood and all organs and tissues such as liver, kidneys and bone was noticeably high at the day of injection. Biodistribution of [^99m^Tc]Tc-AC12-GGGC demonstrated tumour uptake was 2.11 ± 0.46 %ID/g only 2 h after injection. There was significantly lower bone uptake (0.036 ± 0.006 %ID/g) for [^99m^Tc]Tc-AC12-GGGC than for ^89^Zr-labelled DS-5573a (~5% ID/g) at the day of the injection. There was very quick clearance of radioactivity from organs and tissues especially from the blood for [^99m^Tc]Tc-AC12-GGGC. This resulted in significantly (*p* < 0.05) higher tumour-to-blood ratio at 4 h in comparison with 2 h after injection. Importantly, significantly (*p* < 0.05) higher tumour-to-liver and tumour-to-bone ratios were observed at 2 h than 4 h after injection. Thus, the new targeting agent, [^99m^Tc]Tc-AC12-GGGC, provided significantly higher tumour-to-blood ratio (8.18 ± 1.85) only 2 h after injection compared with antibody-based tracer ^89^Zr-DS-5573a (0.12 ± 0.02) resulting in appreciably higher imaging contrast. Taking this into account, [^99m^Tc]Tc-AC12-GGGC could provide better biodistribution and targeting properties for imaging of B7-H3 at day of injection in comparison with the studied antibody-based tracer.

It has to be noted that both tumor-to-organ ratios and the absolute tumor uptake values are important for clinical imaging. The absolute uptake of [^99m^Tc]Tc-AC12-GGGC in the preclinical xenograft model, 2.1 ± 0.4 %ID/g is not very high, which might be considered as an obstacle for clinical translation. On the other hand, the average tumour uptake of [^111^In]In-DTPA-octreotide in the murine models (AR42J and NCI-H69 xenografts) was in the range from 2.5 to 4.2 %ID/g at 3–4 h after injection [57,58], i.e., close to the uptake of [^99m^Tc]Tc-AC12-GGGC. Still, [^111^In]In-DTPA-octreotide was used routinely for clinical visualization of neuroendocrine tumors. Thus, the tumor uptake of ^99m^Tc-labeled AC12-GGGC might be satisfactory for clinical use. However, an affinity maturation of AC12 might be advisable for further clinical development.

## 5. Conclusions

In conclusion, the use of an Affibody-based tracer containing peptide-based cysteine-containing chelator at the C-terminus, provides labelling of the anti-B7-H3 AC12 Affibody molecule with ^99m^Tc. The newly introduced Affibody-based tracer is a potentially suitable for preclinical imaging of B7-H3 expressing tumours at the day of injection. It could be a promising candidate for further development aiming at clinical application in the future.

## Figures and Tables

**Table 1:** Apparent equilibrium dissociation constants (KD) for the interaction between [^99m^Tc]Tc-AC12-GGGC and SKOV-3 cells determined using an InteractionMap analysis of the LigandTracer sensorgrams.

k_a1_ (1/M × s) × 10^4^	k_d1_ (1/s) × 10^−5^	K_D1_ (nM)	Weight_1_, %	k_a2_ (1/M × s) × 10^5^	k_d2_ (1/s) × 10^−3^	K_D2_ (nM)	Weight_2_, %
1.7 ± 0.5	3.0 ± 0.4	1.9 ± 0.8	18.5 ± 4.3	1.2 ± 0.1	8.1 ± 0.5	68.8 ± 7.4	62.9 ± 4.2

**Table 2:** Biodistribution and tumor-to-organ ratio of [^99m^Tc]Tc-AC12-GGGC in BALB/C nu/nu mice bearing SKOV-3 xenografts at 2 and 4 h after injection. Data are expressed as the percentage of administered activity (injected probe) per gram of tissue (%ID/g). The data are presented as the average (*n* = 4) and SD.

Site	Uptake, %ID/g		Tumor-to-Organ Ratio	
	2 h	4 h	2 h	4 h
Blood	0.26 ± 0.01 *	0.10 ± 0.01 *	8.18 ± 1.85 *	11.0 ± 0.53 *
Salivary	0.28 ± 0.01 *	0.15 ± 0.03 *	7.48 ± 1.67	7.06 ± 1.79
Lung	0.35 ± 0.02 *	0.14 ± 0.03 *	5.93 ± 1.13	7.56 ± 1,79
Liver	3.27 ± 0.75	2.57 ± 0.42	0.65 ± 0.12 *	0.41 ± 0.07 *
Spleen	0.82 ± 0.08	0.63 ± 0.11	2.60 ± 0.74	1.70 ± 0.30
Pancreas	0.17 ± 0.02 *	0.06 ± 0.01 *	12.25 ± 1.84	17.33 ± 3.41
Stomach	0.49 ± 0.12 *	0.20 ± 0.05 *	4.75 ± 2.3	5.58 ± 1.55
Small int	0.30 ± 0.07 *	0.16 ± 0.03 *	7.55 ± 2.83	6.54 ± 1.01
Kidney	13.69 ± 1.81	10.30 ± 1.13	0.15 ± 0.02 *	0.10 ± 0.01 *
Tumour	2.11 ± 0.46 *	1.04 ± 0.08 *	-	-
Muscle	0.026 ± 0.006 *	0.014 ± 0.004 *	69.92 ± 19.23	72.16 ± 20.82
Bone	0.036 ± 0.006 *	0.024 ± 0.002 *	58.45 ± 5.00 *	43.55 ± 5.72 *
GI **	1.28 ± 0.16	1.37 ± 0.29	-	-
Carcass **	2.89 ± 0.53	1.39 ± 0.28	-	-

* Significant difference between 2 and 4 h post injection. ** Data for gastrointestinal (GI) tract with content and carcass are presented as % of injected dose per whole sample.

## Data Availability

Data is contained within the article or supplementary material.

## Supplementary Materials

The following supporting information can be downloaded at: https://www.mdpi.com/article/10.3390/pharmaceutics14091780/s1, Figure S1: B7-H3 expression on different cell lines; Figure S2: A. Radiochromatogram of [^99m^Tc]Tc-AC12-GGGC before injection in mice; B and C. Radiochromatograms of urine collected from mice 30 min after injection.
