# Supplementary material for: Evaluation of an Affibody-Based Binder for Imaging of Immune Check-Point Molecule B7-H3
Source: Pharmaceutics. 2022 Aug 25;14(9):1780. doi: 10.3390/pharmaceutics14091780 (PMC9506244; doi:10.3390/pharmaceutics14091780)
Supplement: Supplementary file 1 [file pharmaceutics-14-01780-s001.zip › pharmaceutics-1779509-supplementary.pdf]

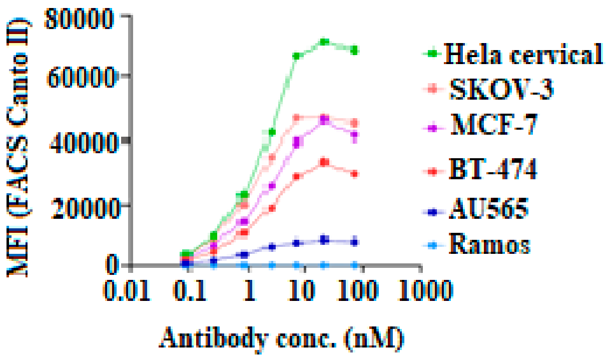

Figure S1. B7-H3 expression on different cell lines.

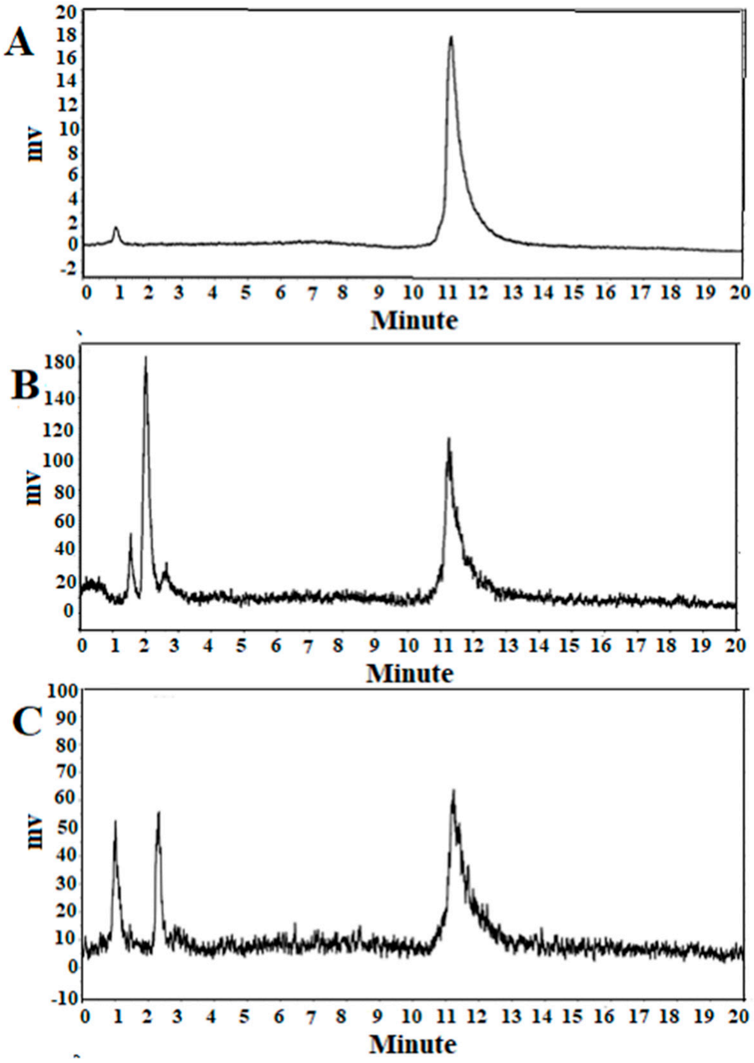

Figure S2. A. Radiochromatogram of [^{99m}Tc]Tc-AC12-GGGC before injection in mice; B and C. Radiochromatograms of urine collected from mice 30 min after injection.
